# Discriminatory capacity of serum interleukin-6 between complicated and uncomplicated acute appendicitis in children: a prospective validation study

**DOI:** 10.1007/s12519-022-00598-2

**Published:** 2022-09-16

**Authors:** Javier Arredondo Montero, Giuseppa Antona, Adriana Rivero Marcotegui, Carlos Bardají Pascual, Mónica Bronte Anaut, Raquel Ros Briones, Amaya Fernández-Celis, Natalia López-Andrés, Nerea Martín-Calvo

**Affiliations:** 1 Pediatric Surgery Department, Hospital Universitario de Navarra, Irunlarrea 3, 31008 Pamplona, Spain; 2 Clinical Analysis Department, Hospital Universitario de Navarra, Pamplona, Spain; 3 Pathology Department, Hospital Universitario de Navarra, Pamplona, Spain; 4 Cardiovascular Translational Research, NavarraBiomed (Miguel Servet Foundation), Hospital Universitario de Navarra, Universidad Pública de Navarra (UPNA), IdiSNA, Pamplona, Spain; 5 School of Medicine, Department of Preventive Medicine and Public Health, University of Navarra, Pamplona, Spain; 6 CIBER Fisiopatología de la Obesidad y Nutrición (CIBERobn), Health Institute Carlos III, Madrid, Spain; 7 Instituto de Investigación Sanitaria de Navarra, IdiSNA, Pamplona, Navarra Spain

**Keywords:** Acute, Appendicitis, Complicated, Diagnostic, Interleukin-6, Pediatric, Uncomplicated

## Abstract

**Background:**

Serum interleukin-6 (IL-6) has a moderate diagnostic performance in pediatric acute appendicitis (PAA). The evidence regarding its capacity to discern between complicated and uncomplicated PAA is scarce.

**Methods:**

We designed a prospective observational study to validate serum IL-6 as a marker for diagnostic classification between complicated and uncomplicated PAA. This study included 205 patients divided into three groups: (1) patients who underwent major outpatient surgery (*n* = 57); (2) patients with non-surgical abdominal pain (NSAP) in whom the diagnosis of PAA was excluded (*n* = 53), and (3) patients with a confirmed diagnosis of PAA (*n* = 95). The PAA patients were further classified as uncomplicated or complicated PAA. IL-6 concentration was determined in all patients at diagnosis. Comparative statistical analysis was performed using the Mann-Whitney *U* test, the Fisher exact test and the Kruskall Wallis test. The area under the receiver operating characteristic curves (AUC) were calculated.

**Results:**

Median (interquartile range, IQR) serum IL-6 values were 2 pg/mL (2.0–3.4) in group 1, 3.9 pg/mL (2.4–11.9) in group 2, and 23.9 pg/mL (11.1–61.0) in group 3 (*P* < 0.001). Among the participants in group 3, those with uncomplicated PAA had median (IQR) serum IL-6 values of 17.2 pg/mL (8.5–36.8), and those with complicated PAA had 60.25 pg/mL (27.1–169) serum IL-6 (*P* < 0.001). At the cut-off point of 19.55 pg/mL, the AUC for the discrimination between patients in group 2 vs. 3 was 0.83 [95% confidence interval (CI) 0.76–0.90], with a sensitivity of 61.3% and a specificity of 86.8. The AUC for the discrimination between patients with uncomplicated and complicated PAA was 0.77 (95% CI 0.68–0.86) and the cut-off point was 25.90 pg/mL, with a sensitivity and specificity of 84.6% and 65.6%, respectively.

**Conclusions:**

Serum IL-6 has a good performance in discerning between complicated and uncomplicated PAA. A score including clinical and radiological variables may increase the diagnostic performance of this molecule.

**Supplementary Information:**

The online version contains supplementary material available at 10.1007/s12519-022-00598-2.

## Introduction

Pediatric acute appendicitis (PAA) is the most frequent urgent abdominal surgical pathology in the world [1, 2]. Despite its high incidence and available clinical experience, PAA continues to constitute an important therapeutic and diagnostic challenge. Routine laboratory tests in emergency departments (ED) show only a moderate yield. In addition, the use of ionizing radiological studies in particularly vulnerable population as children is highly discouraged. Therefore, the radiological test of choice for diagnosis of PAA in children is abdominal ultrasound, which shows a highly variable diagnostic yield owing to operator dependence [3].

Although there are multiple classifications applicable to PAA, the one with the greatest clinical interest is the classification used to distinguish between complicated and uncomplicated PAA owing to its surgical and prognostic implications. The existing literature on the early diagnosis of PAA is focused mostly on the discriminatory capacity of different biomarkers and ratios to distinguish between PAA and non-surgical abdominal pain (NSAP), and the results are inconsistent [4–6]. Compared with patients with uncomplicated PAA, those with complicated PAA show higher serum values of C-reactive protein (CRP), procalcitonin (PCT) [7], calprotectin and total serum bilirubin [8, 9]. However, the evidence regarding the capacity of more recent serum biomarkers, including interleukin-6 (IL-6), to discriminate between complicated and uncomplicated PAA is scarce.

IL-6, a proinflammatory cytokine secreted by macrophages, T cells, endothelial cells, and fibroblasts, is part of the human innate or humoral defense system. It is secreted in response to pathogen-associated molecular patterns and is responsible for stimulating the synthesis of acute phase reactants, such as CRP. At the biological level, IL-6 acts as an endogenous pyrogen, activates adrenocorticotropic hormone production, and modulates hematopoiesis. IL-6 is known to be elevated in a multitude of pathological circumstances, especially in severe bacterial infections and sepsis in both adult and pediatric populations [10, 11].

The diagnostic performance of IL-6 in the context of PAA has been assessed widely. A recent systematic review concluded that the discriminatory capacity of IL-6 to distinguish between PAA and NSAP was only moderate, but the authors also reported that serum level of IL-6 seemed to be correlated with the time of clinical evolution of the appendicitis [12]. Some of the studies included in that systematic review already suggested that IL-6 may be valid to discriminate between complicated and uncomplicated PAA, but those results were hampered by small sample sizes and other limitations associated with study designs. The aim of our study was to analyze the diagnostic performance of serum IL-6 in discriminating between uncomplicated and complicated PAA using a large, prospective cohort of children from the Mediterranean area to address most of the limitations of previous studies.

## Methods

### Study design

This was a prospective, observational study to assess the ability of serum IL-6 to discriminate between complicated and uncomplicated PAA. Three groups of pediatric patients were included in this study: (1) healthy volunteers who came to the hospital for major ambulatory surgery; (2) patients with non-surgical abdominal pain (NSAP) (patients who were initially evaluated with a clinical suspicion of acute abdomen and in whom the presence of urgent abdominal surgical pathology was excluded), and (3) patients with anatomopathological confirmed diagnosis of PAA. Patients in group 3 were further stratified based on histopathologic analysis of the appendix. Congestive, phlegmonous or suppurative appendicitis were considered as uncomplicated appendicitis, and gangrenous or perforated appendicitis were considered as complicated appendicitis. Sociodemographic, clinical, analytical, surgical, radiological and histological variables of all patients were extracted from participants’ clinical records.

Patients were recruited in the Emergency Department and in the Pediatric Department of our hospital when the personnel conducting the investigation were present. The recruitment period extended from February to December 2021. Inclusion and exclusion criteria are shown in Supplementary file 1.

All patients in group 2 were contacted two weeks after their inclusion in the study to ensure that they had not been diagnosed with PAA in that period. All patients in group 3 were followed up on an outpatient basis for one month after the intervention.

### Sample collection

A venous blood sample was obtained from each patient in a vacutainer tube with separator gel (3.5 mL). In patients in group 1 this sample was obtained prior to the scheduled intervention. In patients in groups 2 and 3 blood sample was obtained at the time of inclusion in the study, during their stay in the Emergency Department.

### Measurement of interleukin-6 levels

Serum samples were processed by laboratory personnel blinded to the patient’s group. IL-6 determinations were made by a chemiluminescent assay on an Immulite 2000 XPi analyzer (Siemens Heathineers, Germany). CRP and PCT were determined by chemiluminescent assay on an Alinity-CI analyzer (Abbott Diagnostics, US). All the markers were measured in the same sample concurrently.

### Statistical analysis

For descriptive purposes we used means and standard deviations or the medians and interquartile ranges (IQR) for quantitative variables and proportions for categorical ones. Kolmogorov-Smirnov test was used to assess the normality of quantitative variables. Sociodemographic and clinical variables were compared between groups using the Fisher exact test, the Mann-Whitney *U* test, and the Kruskall Wallis test. The discriminatory capacity of IL-6 was estimated with the area under the receiver operating characteristic curves (ROC). These analyses were performed for the following comparisons: (1) the groups of patients with NSAP vs. the patients with PAA; (2) the group of ambulatory patients vs. the patients with PAA; (3) the group of ambulatory patients and those with NSAP vs. the patients with PAA, and (4) patients with complicated vs. uncomplicated PAA. Additionally, the distance on the ROC curve of each IL-6 value was calculated as the square root of [(1 − sensitivity)^2^ + (1 − specificity)^2^]. The IL-6 value with the shortest distance on the ROC curve was considered the optimal cut-off. The cut-off with the best percentage of correctly classified patients and the cut off with an associated sensitivity of 100% also were included. In further analyses, we performed the Pearson's correlation test to analyse the correlation between serum levels of IL-6 and set of clinical variables. Statistical significance was settled in a *P*-value < 0.05. Statistical analysis was performed with STATA 15.0 (StataCorp LCC).

### Research ethics board committee

This study was approved by our center's clinical research ethics committee prior to December 18, 2020, under code PI_2020/112. The ethical principles of the Declaration of Helsinki were applied for the conduct of this research study. The parents or legal representative of all participants signed an informed consent form prior to the inclusion in the study.

## Results

### Demographic characteristics

This study included 205 patients, divided into three groups: (1) patients who underwent major outpatient surgery (*n* = 57); (2) patients with non-surgical abdominal pain in whom the diagnosis of PAA was excluded (*n* = 53), and (3) patients with a confirmed diagnosis of PAA (*n* = 95). Significant differences between groups were observed for age, sex, weight, and body mass index. Participants’ sociodemographic characteristics by group are shown in Table 1.

**Table 1:** Sociodemographic characteristics of the patients

Sociodemographics	Group 1 (ambulatory controls) *n* = 57	Group 2 (NSAP) *n* = 53	Group 3 (PAA) *n* = 95	Total	*P*-value
Age, y	8.54 (3.25)	11.14 (2.48)	9.74 (3.26)	9.77 (3.20)	< 0.001
Sex, M/total	43/57 (75.4%)	25/53 (47.16%)	63/95 (66.31%)	131/205 (63.9%)	0.007
Weight, kg	34.15 (16.97)	45.82 (15.61)	36.48 (13.74)	38.25 (15.79)	< 0.001
Body mass index, kg/m^2^	20.48 (8.18)	20.53 (3.94)	15.55 (3.00)	18.78 (3.90)	< 0.001

Numbers are mean (standard deviation) or number (percentage)

*NSAP* non-surgical abdominal pain, *PAA* pediatric acute appendicitis, *M* male

### Clinical and analytical characteristics

**Fig. 1:**
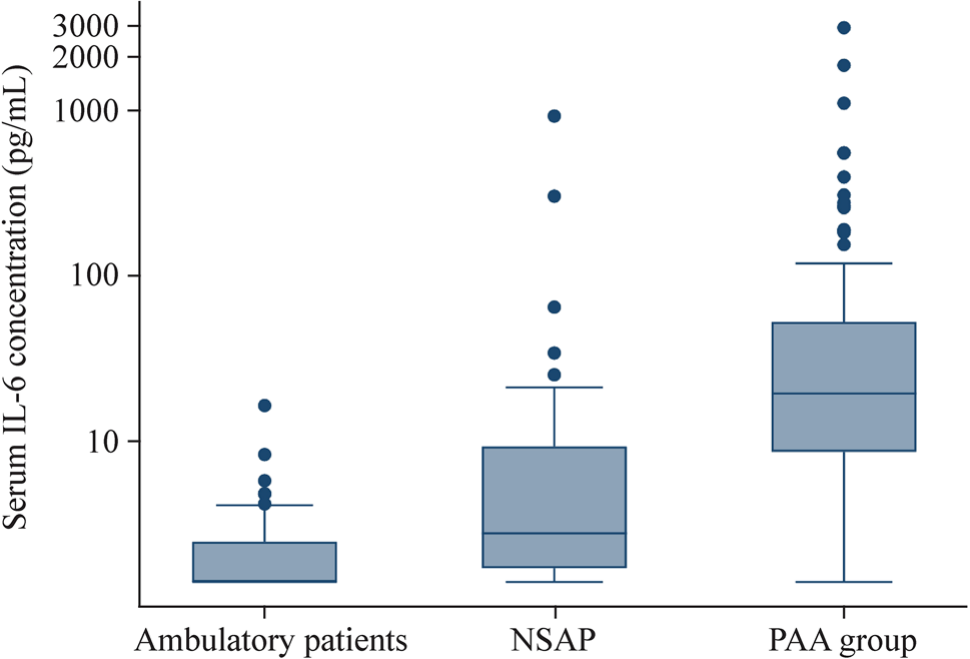
Algorithmic box-plot representation of interleukin-6 (IL-6) serum concentrations in the three study groups. *NSAP* non-surgical abdominal pain, *PAA* pediatric acute appendicitis

Median (IQR) serum IL-6 values were 2 pg/mL (2–3.4 pg/mL) in group 1, 3.9 pg/mL (2.4–11.9 pg/mL) in group 2, and 23.9 pg/mL (11.1–61 pg/mL) in group 3 (*P* < 0.001). The graphical representation of IL-6 serum values in groups 1, 2 and 3 is shown in Fig. 1. A logarithmic scale was used because of the wide analytical range obtained in the determinations.

**Fig. 2:**
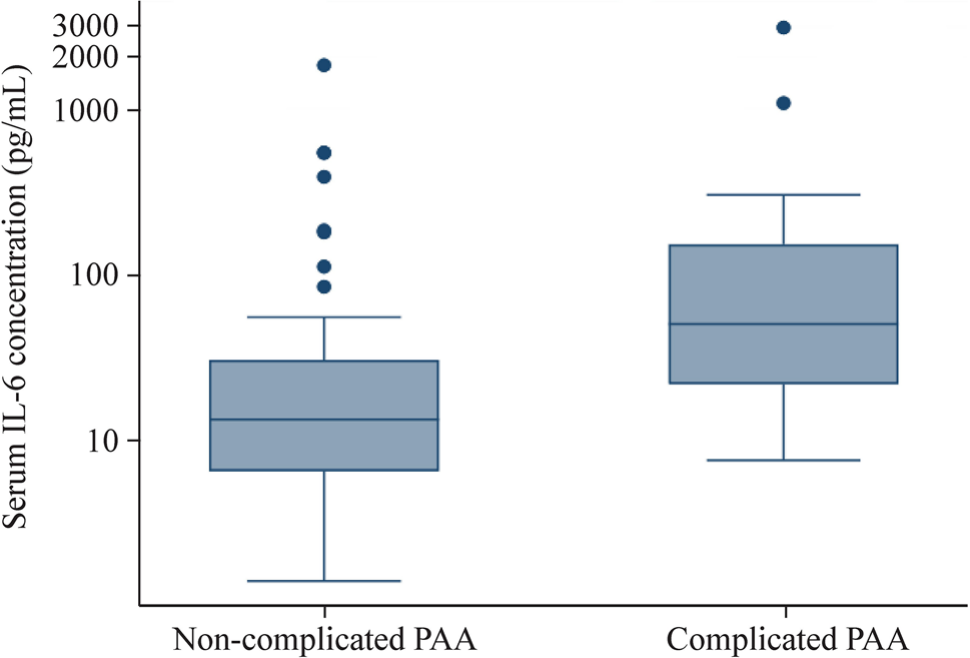
Algorithmic box-plot representation of interleukin-6 (IL-6) serum concentrations in the uncomplicated pediatric acute appendicitis (PAA) and complicated PAA groups

Clinical characteristics of the two main groups of interest (uncomplicated and complicated PAA) are shown in Table 2. Patients with complicated PAA were more likely to present fever and had higher values of total leucocytes, neutrophils, and serum CRP, PCT and IL-6. More specifically, median (IQR) serum value of IL-6 was 17.2 pg/mL (8.5–36.8 pg/mL) in the uncomplicated PAA group and was 60.25 pg/mL (27.1–169 pg/mL) in the complicated PAA group (*P* < 0.001). The graphical representation of IL-6 serum values in uncomplicated PAA and complicated PAA is shown in Fig. 2.

**Table 2:** Clinical characteristics of non-complicated and complicated PAA groups

Clinical variables	Uncomplicated PAA (*n* = 65)	Complicated PAA (*n* = 30)	*P*-value
Hours of pain evolution, h	25.41 (19.72)	30.98 (20.10)	0.10
Fever > 37.8, yes/no/missing	14/47/4 (22.95%)	15/12/3 (55.55%)	0.001
Number of diarrheal stools	0.72 (2.78)	0.50 (1.45)	0.77
Urinary symptoms, yes/no/missing	13/48/4 (21.31%)	7/20/3 (25.92%)	0.60
Number of emetic episodes	2.22 (2.23)	2.70 (2.70)	0.26
Hyporexia, yes/no	48/11 (81.35)	21/5 (80.76)	0.78
Leucocytes (1 × 10^9^/L)	15.14 (4.77)	18.33 (4.59)	0.004
Neutrophils (1 × 10^9^/L)	12.22 (4.75)	15.32 (4.46)	0.003
CRP (mg/L)	31.75 (39.48)	74.60 (70.19)	< 0.001
PCT (ng/mL)	1.00 (6.16)	2.45 (5.14)	< 0.001
IL-6 (pg/mL)*	17.20 (8.50–36.8)	60.25 (27.10–169)	< 0.001

Numbers are mean (standard deviation) or number (percentage)

*PAA* pediatric acute appendicitis, *CRP* C-reactive protein, *PCT* procalcitonin, *IL-6* interleukin-6

“*”Median, interquartile range

**Fig. 3:**
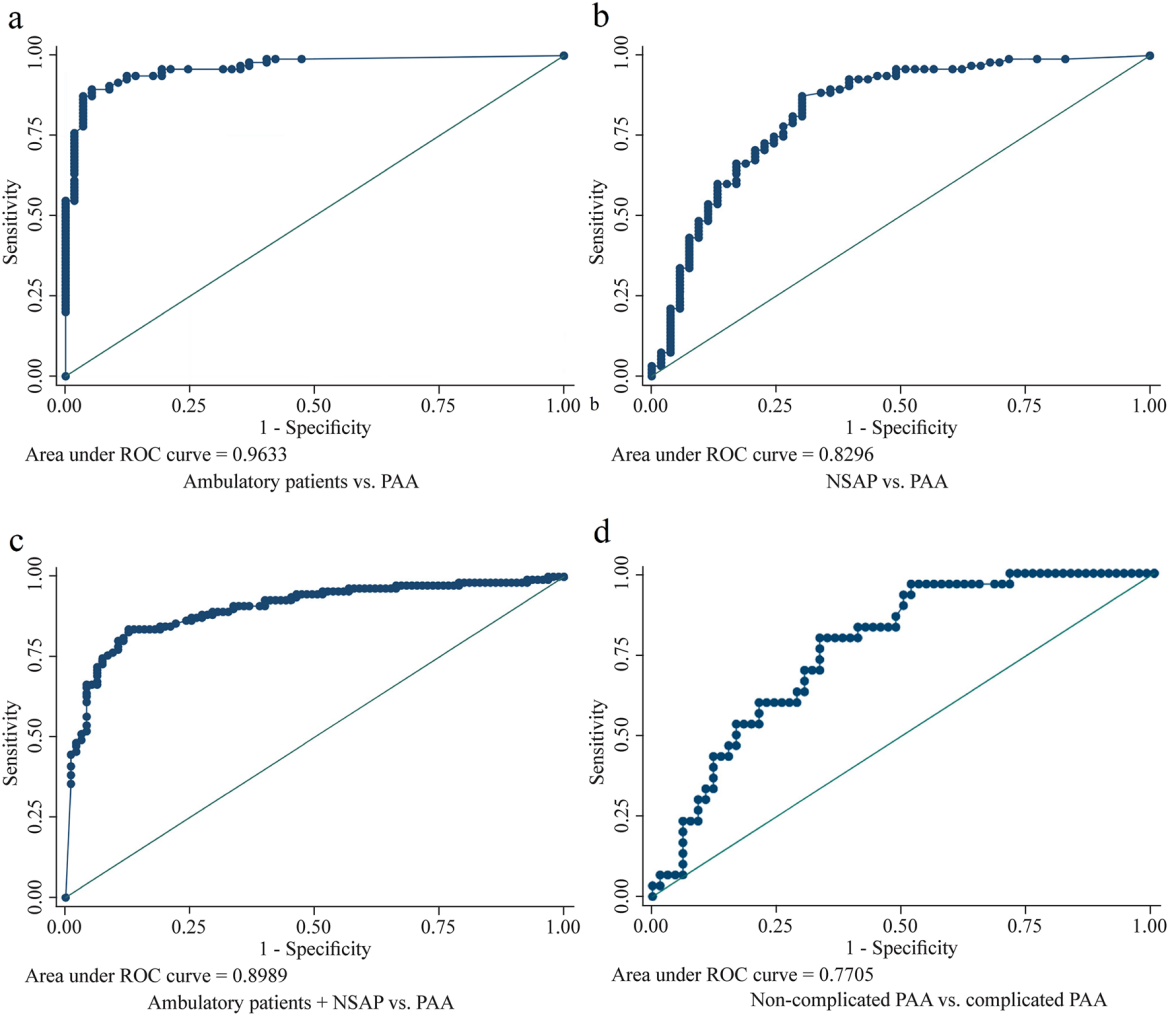
Receiver operating characteristic curves **(**ROC) for interleukin-6 (IL-6) in subgroup analyses. **a** Ambulatory patients vs. PAA; **b** NSAP vs. PAA; **c** ambulatory patients + NSAP vs. PAA; **d** uncomplicated PAA vs. complicated PAA. *NSAP* non-surgical abdominal pain, *PAA* pediatric acute appendicitis

Regarding the capacity of IL-6 to discriminate between patients from groups 2 vs. 3, we found an area under the curve (AUC) of 0.83 [95% confidence interval (CI) 0.76–0.90] (*P* = 0.037). The cut-off point with the shortest distance on the ROC curve resulted in 19.55 pg/mL, with a sensitivity of 61.3% and a specificity of 86.8%. A higher value of AUC was obtained for the discrimination between patients in groups 1 vs. 3 (AUC: 0.96; 95% CI 0.93–0.99) (*P* = 0.013). In this analysis, the cut-off point was 20.70 pg/mL, with a sensitivity of 54.7% and a specificity of 100%. In further analysis we calculated the AUC for the discrimination between patients from groups 1 and 2 vs. patients in group 3 and obtained an AUC of 0.90 (95% CI 0.86–0.94) (*P* < 0.001). In this analysis the cut-off point was 18.6 pg/mL, with a sensitivity of 58.9% and a specificity of 92.7%. Lastly, we performed the ROC analysis comparing uncomplicated vs. complicated PAA and obtained an AUC of 0.77 (95% CI 0.68–0.86) (*P* = 0.051). The cut-off point with the shorter ROC distance was 25.90 pg/mL, with a sensitivity and specificity of 84.6% and 65.6%, respectively (Table 3). The graphical representation of the different ROC curves is shown in Fig. 3.

**Table 3:** Diagnostic performance of serum interleukin-6 (IL-6) in different stratified analyses

Group comparison	AUC value	95% CI	Proposed IL-6 cut off (pg/mL)	*P*-value	Sensitivity (%)	Specificity (%)
Ambulatory patients vs. PAA	0.96	0.93–0.99	20.70	0.01	54.7	100.0
NSAP vs. PAA	0.83	0.76–0.90	19.55	0.04	61.3	86.8
Ambulatory patients + NSAP vs. PAA	0.90	0.86–0.94	18.60	< 0.001	58.9	92.7
Non-complicated PAA vs. complicated PAA	0.77	0.68–0.86	25.90	0.05	84.6	65.6

*AUC* Area under the curve, *CI* confidence interval, *NSAP* non-surgical abdominal pain, *PAA* pediatric acute appendicitis, *IL-6* interleukin-6

Additionally, we evaluated alternative serum IL-6 cut-off values for discrimination between complicated and uncomplicated PAA and assessed their diagnostic performance in terms of sensitivity and specificity (Table 4). The highest percentage of correctly classified participants (74%) was found at the cut-off points of 58.7 and 66 pg/mL.

**Table 4:** Proposed alternative cut-offs for serum interleukin-6 (IL-6), uncomplicated pediatric acute appendicitis (PAA) vs complicated PAA

IL-6 cut-off value (pg/mL)	Correctly classified (%)	Sensitivity (%)	Specificity (%)
9.8	51.04	100.0	28.8
25.9	71.30	84.6	65.6
58.7	74.00	53.3	83.3
66.0	74.00	43.3	87.9

In further analyses, we performed a set of correlation analyses between serum IL-6 values and the following clinical variables: patients' hours of clinical evolution (*r* = 0.09) (*P* = 0.25), axillary temperature at admission (*r* = 0.30) (*P* = 0.0002), serum CRP (*r* = 0.15) (*P* = 0.05), PCT (*r* = 0.14) (*P* = 0.11), serum neutrophils (*r* = 0.24) (*P* = 0.003) and total serum leukocytes (*r* = 0.21) (*P* = 0.009). All the correlations were weak, but those for axillary temperature at admission, serum CRP, PCT, serum neutrophils and total serum leucocytes were statistically significant.

## Discussion

The pathophysiology of PAA has not been fully elucidated to date. It is known that luminal obstruction of the appendix (e.g., due to the presence of appendicoliths) leads to an increase in intraluminal pressure and that when this pressure increase is equal to the tissue perfusion pressure, tissue ischemia, gangrene, and perforation may occur [13]. However, luminal obstruction of the appendix is not documented in all cases; so this etiology does not explain all diagnosed cases of PAA. Another possible explanation is the presence of a progressive underlying inflammatory process, which also can lead to the same sequence of increased intraluminal pressure, ischemia, gangrene and perforation [13]. In relation to this inflammatory process, bacterial, viral or parasitic focal infectious diseases have been postulated as a possible etiopathogenic origin, although scientific evidence is scarce [14–16]. Other etiologies have been described, such as neoplastic or traumatic, but they are rare [17, 18].

This study with 205 patients is the first prospective study analysing the capacity of IL-6 to discriminate between uncomplicated and complicated PAA. Considering our results, we think that serum values of IL-6 could be used either part as a score including other variables, such as axillary temperature, serum neutrophil, serum total leukocyte counts, CRP and PCT, to help make a rapid identification of children at risk of having a complicated PAA.

We found that IL-6 had a moderate diagnostic performance in distinguishing PAA from NSAP, which is consistent with the existing literature. The major advantages of our study are prospective design and large sample size. In addition, we used a sample of Spanish children, for whom the diagnostic performance of IL-6 in PAA had not been analysed previously. Therefore, our findings confirmed the external validity of previous studies and concluded that the diagnostic performance of IL-6 was similar in children from the Mediterranean area.

Our findings regarding the discrimination between complicated and uncomplicated PAA are not surprising considering that evolved appendicitis is more likely to present bacterial translocation, peritoneal contamination, bacteraemia and sepsis. Nevertheless, our results add to the existing literature because we calculated the cut-off point with the shortest ROC distance in our sample and evaluated the validity of alternative cut-off points that could be used when higher sensitivity or specificity is desired.

In this work, we obtained direct correlations between serum values of IL-6 and other analytical biomarkers, such as CRP, PCT, serum neutrophils and serum total leukocytes. Despite the biological mechanism by which CRP synthesis is directly stimulated by IL-6, we found only a weak correlation (*r* = 0.15, *P* = 0.05). We attribute this finding to the wide range of IL-6 serum values in the context of PAA.

Surprisingly, the weakest correlation we calculated was the one comparing IL-6 serum values with the time of clinical evolution (*r* = 0.09, *P* = 0.25). Although it is known that IL-6 presents, from the pathophysiological point of view, an elevation peak in the first hours after the onset of the inflammatory/infectious event, in the case of PAA we believe this finding is more variable for two reasons: first, because the onset of the process is focal and can take a variable time to have systemic repercussions (not all patients have the same degree of containment of the process or the same rate of disease progression), and second, because there is important variability in the clinical presentation between patients, the hours of clinical evolution being sometimes an inaccurate parameter of the real time of evolution of the disease. In a recently published systematic review regarding the diagnostic performance of IL-6 in PAA, IL-6 serum values seemed to increase with the hours of evolution of the disease, although the evidence obtained was low [12]. We would like to highlight that in our series the analyses did not show the presence of a significant IL-6 peak in the first hours that followed the onset of symptoms.

One important aspect to consider when assessing the validity of a diagnostic tool is the cost attributed to each determination, both in economic terms and in terms of human resources. IL-6 determination is not routinely available in our pediatric emergency department, but it is available on a scheduled basis. The processing time in our laboratory is approximately 60 minutes, and the estimated cost per determination is 8.9 euros (URV, 2020), with a theoretical cost of 5.30 euros (excluding taxes). These statistics are similar with those reported in a systematic review and cost-trade off analysis published in 2017 in terms of economics (15.5 £ per determination), but not in terms of time (168 hours per determination) [19].

Our study has several strengths including prospective design, large sample size and thorough analyses. The main weakness of our study is the convenient sampling design, which is susceptible to selection bias. Nevertheless, because inclusion and exclusion criteria were strictly applied, we do not think that our findings could be explained by selection bias.

In conclusion, we believe that in the current scenario, in which conservative management of PAA is increasingly important, it is essential to have diagnostic elements that allow a rapid identification of children at risk of complicated PAA. IL-6 alone has a moderate discriminatory capacity to distinguish complicated from uncomplicated PAA. We believe that it would benefit from inclusion in a multivariate score that includes other clinical, analytical and radiological parameters to improve its diagnostic performance and to provide a tool which is applicable in routine clinical practice.

## Supplementary Information

Below is the link to the electronic supplementary material.

Supplementary file1 (DOCX 15 KB)

## Data Availability

All data pertaining to this study are available upon justified request through the author in correspondence.
