# Supplementary material for: Discriminatory capacity of serum interleukin-6 between complicated and uncomplicated acute appendicitis in children: a prospective validation study
Source: World J Pediatr. 2022 Sep 16;18(12):810–7. doi: 10.1007/s12519-022-00598-2 (PMC9617836; doi:10.1007/s12519-022-00598-2)
Supplement: Supplementary file 1 — Supplementary file1 (DOCX 15 KB) [file 12519_2022_598_MOESM1_ESM.docx]

**Supplementary file 1. Inclusion and exclusion criteria**

**Inclusion criteria**

The study will include patients aged 0 to 14 years inclusive who present at the pediatric emergency department with acute abdominal pain suggestive of acute appendicitis (initially mesogastric pain and later radiating to the right iliac fossa, pain starting in the right iliac fossa) of less than 5 days of evolution and associated with at least one of the following symptoms: hyporexia, nausea, vomiting, febrile fever, fever, hyporexia, diarrhea. Inclusion in the group of cases will be confirmed with the anatomopathological diagnosis of the surgical specimen.

**Exclusion criteria**

- Patients with a clear suspicion of acute appendicitis or with clinical instability who do not require complementary tests prior to surgery.

- Patients with metastatic neoplasia.

- Patients with hematological alterations.

- Patients with active autoimmune disorders.

- Patients previously appendectomized.

- Patients who have been treated with immunosuppressants in the 28 days prior to the evaluation in the ED.

- Patients who have been treated with systemic steroids in the 14 days prior to the emergency evaluation.

- Patients who have had abdominal trauma prior to ED evaluation.
